# The role of TGF‐β1/Smad2/3 pathway in platelet‐rich plasma in retarding intervertebral disc degeneration

**DOI:** 10.1111/jcmm.12847

**Published:** 2016-04-06

**Authors:** Huilin Yang, Chenxi Yuan, Chunshen Wu, Jiale Qian, Qing Shi, Xuefeng Li, Xuesong Zhu, Jun Zou

**Affiliations:** ^1^ Department of Orthopaedic SurgeryThe First Affiliated Hospital of Soochow UniversitySuzhouJiangsuChina

**Keywords:** platelet‐rich plasma, TGF‐β1/Smad2/3, intervertebral disc degeneration, rabbit, nucleus pulpous

## Abstract

Recent studies have suggested that platelet‐rich plasma (PRP) injections are an effective way to retard intervertebral disc degeneration, but the mechanism of action is unclear. Activated platelets release some growth factors, such as transforming growth factor‐β1 (TGF‐β1), which positively modulate the extracellular matrix of nucleus pulposus cells. The purpose of this study was to explore the mechanism underlying the PRP‐mediated inhibition of intervertebral disc degeneration. In an *in vitro* study, we found that the proliferation of nucleus pulposus cells was greatly enhanced with 2.5% PRP treatment. The TGF‐β1 concentration was much higher after PRP treatment. PRP administration effectively increased the collagen II, aggrecan and sox‐9 mRNA levels and decreased collagen X levels. However, Western blotting demonstrated that specifically inhibiting TGF‐β1 signalling could significantly prevent nucleus pulpous cellular expression of Smad2/3 and matrix protein. In a rabbit study, magnetic resonance imaging revealed significant recovery signal intensity in the intervertebral discs of the PRP injection group compared with the very low signal intensity in the control groups. Histologically, the PRP plus inhibitor injection group had significantly lower expression levels of Smad2/3 and collagen II than the PRP group. These results demonstrated that a high TGF‐β1 content in the platelets retarded disc degeneration *in vitro* and *in vivo*. Inhibiting the TGF‐β1/Smad2/3 pathway could prevent this recovery by inactivating Smad2/3 and down‐regulating the extracellular matrix. Therefore, the TGF‐β1/Smad2/3 pathway might play a critical role in the ability of PRP to retard intervertebral disc degeneration.

## Introduction

Intervertebral disc degeneration is considered a chief cause of low back pain 1. However, its pathogenesis and exact mechanisms remain unknown. With the recent developments in molecular biology, researchers have investigated the mechanisms of intervertebral disc degeneration and evaluated many biological therapies at the cellular, molecular and genetic levels to establish safe and effective clinical therapies for the treatment of disc degeneration diseases.

Platelet‐rich plasma (PRP), which can be prepared by centrifugation, is an autologous blood product that contains a supraphysiological platelet concentration. Since PRP was first used for maxillofacial reconstruction by Whitman *et al*. and Marx *et al*. 2, 3, extensive efforts have been made to expand its role in tissue repair and regeneration, including bone injury 4, wound healing 5, chronic soft tissue injury 6 and cartilage injury 7. Platelet‐rich plasma was able to stimulate chondrocyte proliferation and had a positive clinical effect on knee cartilage degradation 8. Except for some early studies, many have reported its potential effectiveness in treatment. Platelet‐rich plasma has been clinically used for wound healing and tissue regeneration in orthopaedic, oral‐maxillofacial and plastic surgery.

Platelet‐rich plasma has been shown to positively modulate the extracellular matrix of the intervertebral disc in both *in vitro* and animal studies 9, 10. However, the mechanisms involved have not yet been defined. Platelet‐rich plasma contains a multitude of growth factors at concentrations higher than that of baseline blood, including transforming growth factor‐β1 (TGF‐β1). Transforming growth factor‐β1 exists in the highest concentration and shows more important in PRP 11. Therefore, we hypothesized that PRP administration in the intervertebral disc could recover degeneration and that TGF‐β1 signalling may play a positive role in PRP‐mediated processes. This study aimed to provide experimental evidence and an appropriate clinical treatment approach for degenerative intervertebral disc diseases.

## Materials and methods

### Isolation and culture of primary nucleus pulposus cells

All experimental protocols were approved by the Ethics Committee of the First Affiliated Hospital of Soochow University and were performed in accordance with the approved guidelines for the care and use of laboratory animals. Intervertebral discs from three rabbits (2–3 months old) were extracted immediately after killing them. All surgical procedures were performed under sterile conditions. The entire thoracolumbar spine was removed, and the front disc attached to the muscles was stripped and rinsed twice with PBS solution (containing streptomycin 1 g/l and penicillin 1,000,000 U/l). The annulus fibrosus was then incised, and the gelatinous nucleus pulposus was removed. The gelatinous nucleus pulposus was then rinsed twice with DMEM supplemented with F12 (DMEM/F12; Thermo Fisher Scientific, Asheville, NC, USA) and digested in type II collagenase solution (Sigma‐Aldrich, St. Louis, MO, USA) at 37°C for 15–20 min. The digested tissues were pipetted gently, passed through a mesh filter and suspended by centrifugation at 200 × g for 8 min. A total of 1 × 10^5^ cells/ml was seeded into the DMEM/F12 medium and incubated at 37°C under 5% CO_2_ and saturated humidity. When the cell density reached 90%, cells were digested with 0.25% trypsin (Thermo Fisher Scientific) and passaged at a dilution of 1:2.

### Preparation of platelet‐rich plasma

Before isolating nucleus pulposus cells, 10 ml of whole rabbit blood from the same rabbit's central ear artery was extracted and centrifuged at 125 × g for 10 min. Plasma and platelets in the superior layer were collected and centrifuged for another 10 min. at 500 × g The remaining supernatant (approximately 1 ml), including the precipitates, constituted the PRP. The PRP was mixed well and incubated with 110 μl of 10% CaCl_2_ solution containing 100 U of bovine thrombin (Sigma‐Aldrich) at room temperature for 60 min. and then centrifuged at 500 × g for 10 min. The activated PRP‐containing supernatant was collected and stored at −80°C.

### Measurement of TGF‐β1 concentrations

Transforming growth factor‐β1 concentrations were measured using a double antibody sandwich avidin–biotin complex ELISA. To measure the concentrations, ELISA kits for TGF‐β1 (Westang Biotech, Shanghai, China) were used according to the manufacturer's instructions. The absorbance values of the samples were measured at 450 nm using an ELISA plate reader (Model 680; Bio‐Rad, Hercules, CA, USA). The TGF‐β1 concentration was positively proportionate to the absorbance values; therefore, the TGF‐β1 concentrations in the samples were calculated using a standard curve that was plotted as absorbance *versus* TGF‐β1 concentration. All samples and standards were measured in duplicate.

### Determination of cell viability

Passage one nucleus pulposus cells were trypsinized, dispersed into single‐cell suspensions and seeded into 96‐well plates 5 × 10^4^/ml. Each well contained 100 μl of cell suspension. Cell viability in the experimental groups was determined at 1, 2, 3, 4, 5, 6 and 7 days after the induction with 10%, 5%, 2.5% and 1% volume fractions of PRP. The controls were cultured in the regular medium without PRP. The measurement procedure was as follows: 10 μl of Cell Counting Kit‐8 (CCK‐8) solution (Dojindo Molecular Technology, Rockville, MD, USA) was added to each well and the plates were incubated at 37°C and 5% CO_2_ for 1 hr. After the incubation, the supernatants were carefully aspirated. To lyse the cells and completely dissolve the precipitates, dimethyl sulfoxide (DMSO) was added to each well and the plates were shaken for 15 min. The absorbance was then measured at 450 nm. The background absorbance of the medium in the absence of cells was subtracted. The experiments were independently repeated at least three times.

### Identification of related genes using reverse transcription–polymerase chain reaction

Five days after induction with 2.5% PRP, 2.5% PRP + 10 μmol/l TGF‐βl inhibitor SB431542 (Selleck Chemicals, Houston, TX, USA), the nucleus pulposus cells were collected and the total RNA was extracted using TRIzol reagent (Invitrogen, Carlsbad, CA, USA). The cells not subjected to PRP induction were also collected as controls. The RNA samples were quantified and reverse transcribed into first‐strand cDNA. Quantitative polymerase chain reactions (PCR) were performed on a real‐time TP800 system (TaKaRa Bio, Otsu, Shiga, Japan) to detect the genetic expression of the chondrogenic markers collagen II, aggrecan, and sox 9 and hypertrophic marker collagen X. A reference gene (glyceraldehyde‐3‐phosphate dehydrogenase, GAPDH) was amplified in parallel with the target genes. The total volume of the reaction mixture used for each PCR was 20 μl, which included 10 μl of SYBR Green mixture, 1 μl of each primer (10 μmol/l), 1 μl of reverse primer (10 μmol/l), 1 μl of cDNA template and 7 μl of ddH_2_O. The PCR conditions were as follows: 95°C for 10 min., 95°C for 15 sec., 60°C for 30 sec. and 72°C for 30 sec. for 40 cycles. Relative gene expression was normalized against GAPDH expression, and the data were presented as the fold change using the formula 2^−ΔΔCT^, as recommended by the manufacturer. The primer sequences are listed in Table 1.

**Table 1:** Primers used in real‐time polymerase chain reaction

Gene	Primer sequence
Collagen II	Forward primer 5′‐GCTCCCAGAACATCACCTACCA‐3′ Reverse primer 5′‐ACAGTCTTGCCCCACTTACCG‐3′
Aggrecan	Forward primer 5′‐AGGTCGTGGTGAAAGGTGTTGTG‐3′ Reverse primer 5′‐TGGTGGAAGCCATCCTCGTAG‐3′
Sox‐9	Forward primer 5′‐CGAGCCGGACCTCAAGAAG‐3′ Reverse primer 5′‐ GCACCAGCGTCCAGTCGTA‐3′
Collagen X	Forward primer 5′‐GCCCTTCTGCTGCTAGTGTCTTT‐3′ Reverse primer 5′‐TGTGTCTTGGTGTTGGGTTGTG‐3′
GAPDH	Forward primer 5′‐AAGGTCGGAGTGAACGGATTTG‐3′ Reverse primer 5′‐CGTGGGTGGAATCATACTGGAAC‐3′

### Western blot analysis

Nucleus pulposus cells were harvested at 5 days after induction with 2.5% PRP, 2.5% PRP + inhibitor and inhibitor only. Non‐induced cells served as the controls. The cells were washed three times with PBS and suspended in ice‐cold lysis buffer (Bio‐Rad). The lysates were separated by 12% SDS‐PAGE, and proteins were transferred to nitrocellulose membranes. Membranes were blocked with 1× Tris‐buffered saline with Tween‐20 buffer (Thermo Fisher Scientific) containing 5% milk and incubated overnight at 4°C with collagen II (Novus Biologicals, Littleton, CO, USA) and Smad 2/3 (Biosynthesis Biotech, Beijing, China) primary antibodies. The next day, the membranes were washed and incubated with the corresponding peroxidase‐conjugated secondary antibodies at room temperature for 1 hr. The membranes were then developed using an enhanced chemiluminescence detection system. The β‐actin expression level was used as an internal control.

### Experimental animals

New Zealand rabbits (*n* = 24) were randomly divided into three groups. Each of the rabbits weighed 2.5 ± 0.3 kg at the beginning of the study, and all were anaesthetized with 30 mg/kg pentobarbital. After the rabbits were shaved, their skin was sterilized with povidone iodine. The rabbits were then placed in the lateral prone position, and the anterior surfaces of two consecutive lumbar intervertebral discs (L4‐5 and L5‐6) were exposed using a posterolateral retroperitoneal approach. The nucleus pulposus tissues of the intervertebral discs were punctured using an 18G needle on a 5‐ml syringe to induce disc degeneration. Four weeks after the initial puncture, either 15 μl of autologous PRP (drawn from the same rabbit's central ear artery) or 15 μl of PRP + 10 μl of TGF‐βl inhibitor was injected into the centre of the rabbit nucleus pulposus from the contralateral side using a 26G needle with a microsyringe (Hamilton Bonaduz AG, Bonaduz, Switzerland). Other eight rabbits' L4‐5 and L5‐6 discs were approached but not treated and referred to as the ‘control group’. After surgery, all of the rabbits were allowed to move freely in a cage.

### Magnetic resonance imaging

Magnetic resonance imaging (MRI) was performed on each rabbit at 0, 4, 8 and 12 weeks after the operation to assess the degree of disc degeneration. Sagittal T2‐weighted images were obtained using a 0.2 T E‐Scan XQ Extremity MRI (Esaote, Genoa, Italy). In the T2‐weighted images, 16 discs (eight animals) per group were classified according to the modified Thompson classification from grade I to IV (I, normal; II, minimal decrease of signal intensity but obvious narrowing of high signal area; III, moderate decrease of signal intensity; and IV, severe decrease of signal intensity) 12.

### Histological and immunohistochemical analyses

The intervertebral discs were harvested, fixed in 4% paraformaldehyde, decalcified with 10% ethylenediaminetetraacetic acid and embedded in paraffin using standard procedures. The sections were stained with haematoxylin and eosin to evaluate disc degeneration. For immunohistochemistry detection, sections were incubated with primary antibodies specific to collagen II (Novus Biologicals) and Smad2/3 (Biosynthesis Biotech). The obtained images were captured on an image analysis system and analysed by Image Pro Plus 6.0 software (Media Cybernetics, Baltimore, MD, USA). Integrated optical density was calibrated, and the area of interest was established. The mean optical density was defined as the integrated optical density divided by area (mm^2^).

### Statistical analysis

All quantitative data are presented as mean ± S.D. Statistical analyses were performed using one‐way anova. For non‐parametric data, the Kruskal–Wallis test was performed. Differences with values of *P* < 0.05 were considered statistically significant.

## Results

### Morphological observation of rabbit nucleus pulposus cells

Most suspended cells adhered to the dishes in approximately 24 hrs. Within 48 hrs, the adherent cells changed from oval to fusiform or polygonal. The nuclear volume accounted for one third of the total cell volume. Furthermore, the cells showed strong refraction features and were rich in cytoplasm. The cell pseudopodia elongated with increasing culture time. Approximately 9–12 days later, the cells reached 90% confluence and aggregated into spiral or shoal shapes.

### Platelet count in whole blood and PRP

The number of platelets was counted with a haemocytometer. The platelet concentration was 282.25 ± 7.93 × 10^9^/l in whole blood and 1170.25 ± 56.08 × 10^9^/l in PRP. The mean platelet count of the PRP was 4.15 ± 0.28‐fold higher than that of whole blood.

### ELISA for TGF‐β1 concentrations

A standard curve was created based on the absorbance values at 450 nm for standards of different concentrations. Transforming growth factor‐β1 concentrations of whole blood and PRP samples were determined using the equation generated from the standard curve. The TGF‐β1 concentration was 328.56 ± 45.88 pg/ml in whole blood and was 1180.13 ± 45.88 pg/ml in PRP. The TGF‐β1 concentration in PRP was 3.65 ± 0.60‐fold higher than that of whole blood.

### CCK‐8 detection

**Figure 1:**
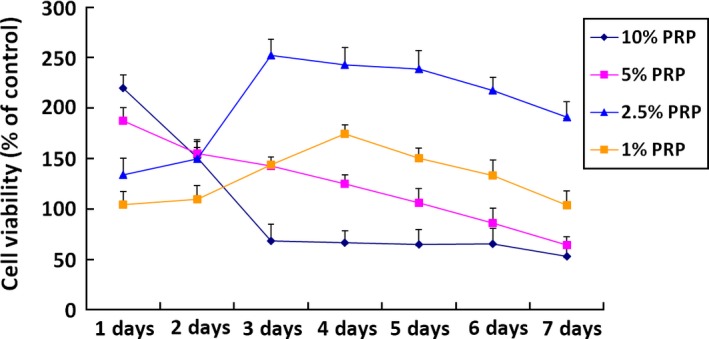
Effects of different concentrations of PRP on nucleus pulposus cell proliferation by CCK‐8 detection. PRP with a 2.5% volume fraction showed significant differences at each time‐point compared to the control group.

To examine whether the effect of PRP on cellular activity was time and dose dependent, we co‐cultured rabbit nucleus pulposus cells with PRP at 10%, 5%, 2.5% and 1% volume fractions and determined cell viability at days 1, 2, 3, 4, 5, 6 and 7 (Fig. 1). The CCK‐8 results showed that high PRP concentrations significantly stimulated rabbit nucleus pulposus cells proliferation on day 1. This effect declined with increasing incubation time, which suppressed cell growth. In comparison, low PRP concentrations had a longer and steadier effect on cell proliferation. Platelet‐rich plasma with a 2.5% volume fraction showed significant differences at each time‐point compared with the control group (*P* < 0.05).

### Real‐time PCR

**Figure 2:**
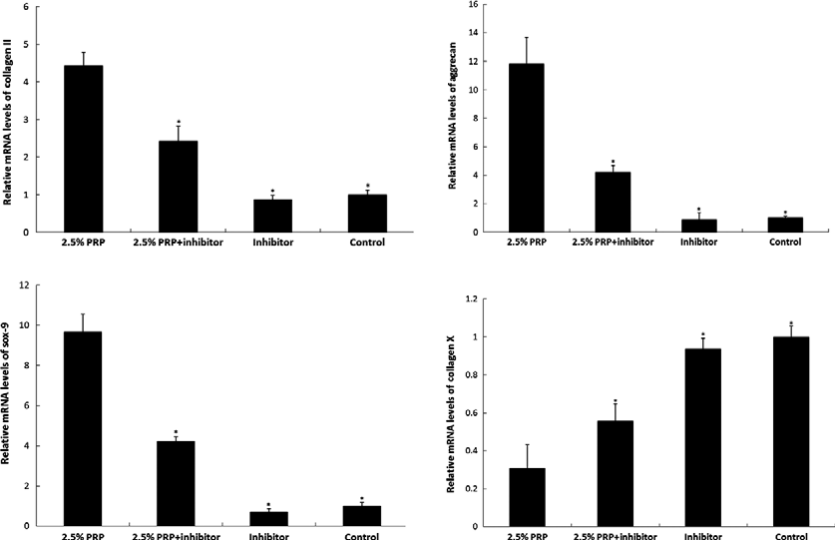
Effect of PRP on related gene expressions *in vitro*. Real‐time reverse transcription–polymerase chain reaction results show that the mRNA expressions of collagen II aggrecan and sox‐9 were significantly increased, whereas collagen X expression was decreased in PRP‐treated nucleus pulposus cells. In contrast, nucleus pulposus cells treated with transforming growth factor‐β1 inhibitor exhibited low expression levels of collagen II, aggrecan and sox‐9 and high collagen X expression (*P < 0.05 compared to the 2.5% PRP group).

We treated rabbit nucleus pulposus cells with 2.5% PRP and the TGF‐β1 inhibitor (SB431542), respectively, dissolved in DMSO. Real‐time PCR (RT‐PCR) results showed that the administration of 2.5% PRP significantly increased the collagen II, aggrecan and sox‐9 mRNA levels and decreased the collagen X level compared with control (*P* < 0.05). In contrast, the nucleus pulposus cells treated with PRP and TGF‐β1 inhibitor exhibited low expression levels of collagen II, aggrecan and sox‐9 mRNA and high expression levels of collagen X mRNA. Significant differences were also observed compared with the PRP group (*P* < 0.05) (Fig. 2).

### Western blot analysis

**Figure 3:**
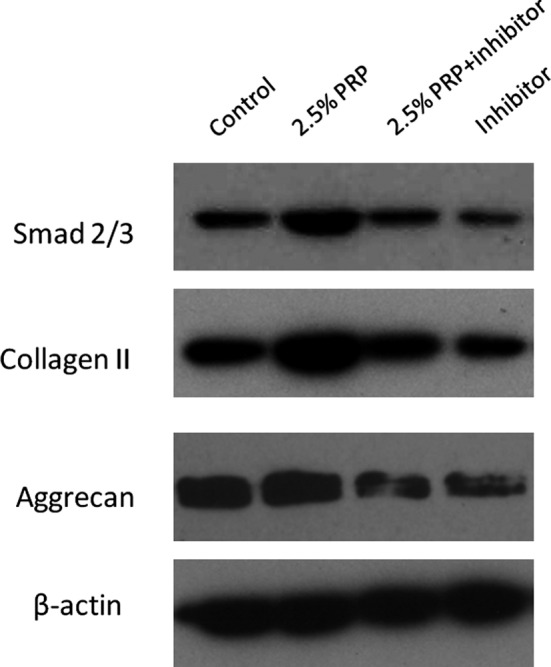
Effect of PRP on functional protein expressions detected using Western blotting. PRP increased Smad2/3 production and collagen II and aggrecan protein levels in the nucleus pulposus cells, but this effect was attenuated by the TGF‐β1 inhibitor.

The Smad2/3 and related nucleus pulposus cells functional protein levels were measured by Western blot analysis; 2.5% PRP treatment significantly increased Smad2/3 production as well as collagen II and aggrecan expressions. However, inhibiting TGF‐β1 receptor activation with TGF‐β1 inhibitor decreased the Smad2/3, collagen II and aggrecan expressions. Together, these results demonstrate that PRP, dependent on nucleus pulposus cells TGF‐β1 receptors, directly activates the TGF‐β1/Smad2/3 pathway, which leads to collagen II and aggrecan production (Fig. 3).

### MRI analyses

**Figure 4:**
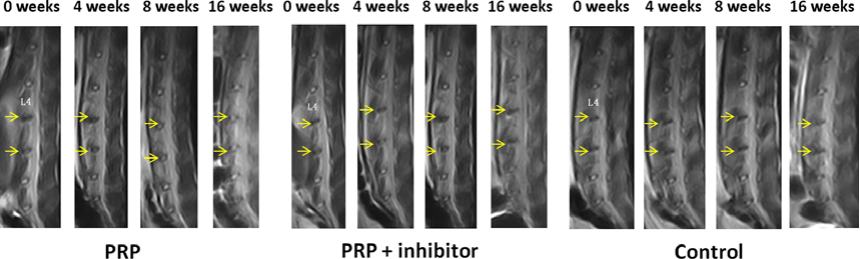
Magnetic resonance imaging findings after PRP injection on rabbit intervertebral discs. T2 signal intensity was stronger for the PRP‐injected discs than for the control discs.

Magnetic resonance imaging was performed at different time‐points after PRP or PRP+TGF‐β1 inhibitor injections (Fig. 4). The T2 signal intensity of the intervertebral discs was significantly higher in the PRP injection group than in the control and PRP + inhibitor groups (*P* < 0.05) (Table 2), which suggested that PRP could reverse the strong T2 signal intensity in intervertebral discs and that the TGF‐β1 inhibitor could suppress the positive effect of PRP on the intervertebral discs.

**Table 2:** Thompson classification for intervertebral discs

Group	Thompson grade				*P*‐value
	I	II	III	IV	
Control	0	0	5	11	0.006
PRP	5	10	1	0	
PRP + inhibitor	0	1	7	8	

### Histological observation of haematoxylin and eosin

**Figure 5:**
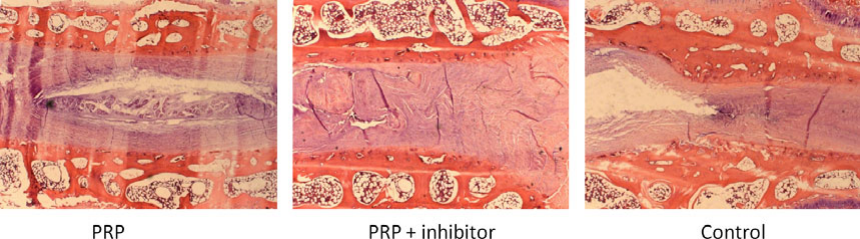
Histological analysis of the intervertebral discs by haematoxylin and eosin staining (×25). Many nucleus pulposus cells were observed in the PRP group. However, some nucleus pulposus cells were replaced with fibrocartilaginous tissue in the two control groups.

The PRP group showed slightly decreased disc height, wrinkled but orderly arranged annulus fibrosus, a blurred boundary between the annulus fibrosus and nucleus pulposus, reduced number of nucleus pulposus cells, fuzzy collagen structure and loss of proteoglycan and water on histological images. However, in the control and PRP+ inhibitor groups, the disc height was severely reduced, the annulus fibrosus was broken, the nucleus pulposus had shrunk and in some cases had even disappeared, the number of cells decreased, and the nucleus pulposus was partially or fully replaced by fibrous‐like tissue (Fig. 5).

### Immunohistochemical analysis

**Figure 6:**
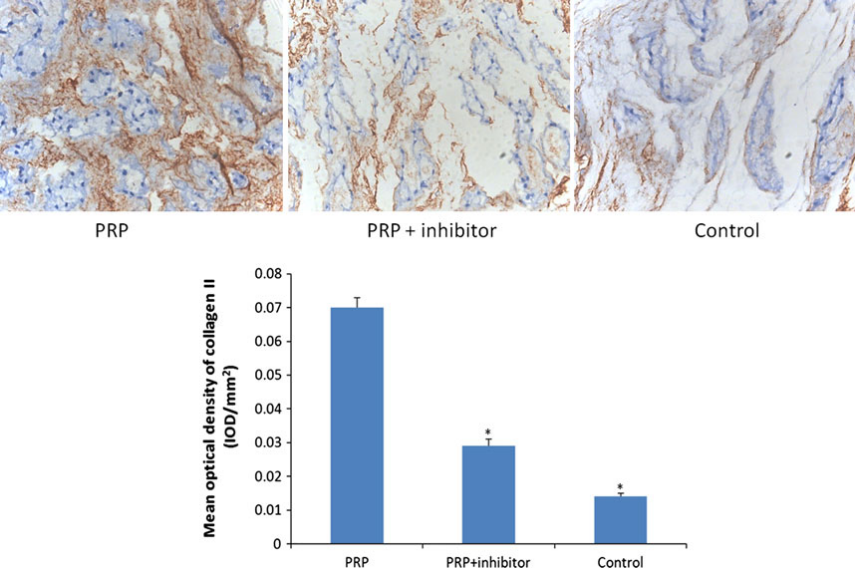
Collagen II immunohistochemical staining (×200). Collagen II, which provides the extracellular matrix framework of cartilage, was detected in PRP group at 8 weeks. Quantification of immunohistochemical staining showed that the PRP‐treated discs had significantly higher mean optical density than the transforming growth factor‐β1 inhibitor–treated and control groups (*P < 0.05 compared to the PRP group).

**Figure 7:**
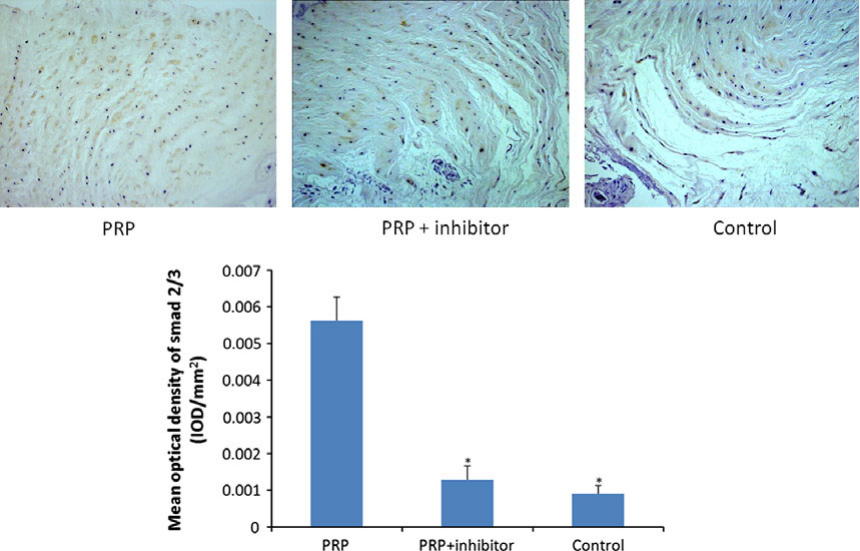
Smad2/3 immunohistochemical staining (×200). Smad2/3 expression was more apparent in PRP group at 8 weeks. Quantification of immunohistochemical staining showed that the PRP group had significantly higher mean optical density than the transforming growth factor‐β1 inhibitor–treated and control groups (*P < 0.05 compared to the PRP group).

To examine the effect of PRP on the intervertebral discs, the collagen II and Smad2/3 expression levels were determined by immunohistochemical analysis. Positive collagen II expression signalling was observed in the PRP group in which the extracellular matrix was deeply stained; however, the staining in the other two groups was slight. Smad2/3‐positive cells appeared brown in the cytoplasm or dark brown in the nucleus under microscopy. The expressions of both collagen II‐ and Smad2/3‐positive cells in the PRP group were statistically higher than those in the control and PRP + inhibitor groups, which were determined by the mean optical density (*P* < 0.05) (Figs 6 and 7).

## Discussion

Platelet‐rich plasma, defined as a sample of autologous blood with platelet concentrations above the baseline values, is prepared by centrifugation. Different centrifugation steps, forces and times could result in different platelet concentrations, activities and yields. In the United States, there are more than 10 different commercialized PRP preparation systems approved by the Food and Drug Administration. In this study, the PRP was prepared after two centrifugations as described by Aghaloo *et al*. 13. According to our results, the percentage of platelets and the concentration of TGF‐β1 in the PRP were 4.15 ± 0.28‐fold and 3.65 ± 0.60‐fold higher, respectively, than those of whole blood.

In this study, we investigated the volume fraction of PRP required to best enhance nucleus pulposus cells proliferation *in vitro*. Several studies showed that the positive effect of PRP on cell proliferation is not dependent on PRP concentration. In some cases, high PRP concentrations could suppress cellular proliferation, whereas low PRP concentrations could have a long‐term positive effect on cellular proliferation. Choi *et al*. reported that the viability and proliferation of alveolar bone cells were suppressed by high PRP concentrations but were stimulated by low PRP concentrations (1–5%) 14. Researchers also demonstrated that higher PRP concentrations may not result in stronger tissue repair. Giusti *et al*. 15 suggested that a compromise between extremely high and low platelet concentrations was required to obtain optimal tendon healing. Platelets have the strongest effect on tissue repair immediately after inflammation; therefore, researchers suggest that a proper time‐point is more important than the concentration. Consistent with a previous study, we found that cell proliferation was most effectively promoted by 2.5% PRP. The exact PRP volume fraction differed from that found in other studies because the cell types used in this study differed, and because PRP content and amount vary among different preparation systems.

Previous researchers have preliminarily shown that PRP promotes nucleus pulposus cells regeneration by the TGF‐β1/Smad signalling pathway, in which Smad2/3 is phosphorylated by TGF‐β1 and released into the cytoplasm, which induces the nucleus pulposus cells re‐differentiation process 16. SB431542 acts as a competitive adenosine triphosphate–binding site kinase inhibitor and has been shown to specifically inhibit the TGF‐β1 receptor, thereby blocking gene expression in the TGF‐β1/Smad2/3 signalling pathway 17. In this study, we showed that PRP administration enhanced nucleus pulposus cells proliferation and the synthesis of chondrogenic markers such as collagen II, aggrecan and sox‐9, while inhibiting hypertrophic marker collagen X expression. However, the TGF‐β1 inhibitor attenuated the effect of PRP. This inhibition resulted in decreased mRNA expression as well as collagen II, aggrecan, and Smad2/3 protein levels, a finding that was consistent with the immunohistochemical detection of reduced collagen II and Smad2/3 levels in the rabbit model. Based on the *in vivo* and *in vitro* results, PRP could retard intervertebral disc degeneration, and the TGF‐β1/Smad2/3 pathway plays a key role in the process.

Intervertebral disc degeneration is characterized by decreased disc height, progressive fraying and dehydration of the nucleus pulposus, resulting in low T2 signal intensity on MRI. In this study, all rabbits were killed 8 weeks after injection, and their intervertebral discs were subjected to sagittal T2‐weighted MRI analysis. We noted an obvious decrease in the signal intensities of the T2‐weighted images of the discs accompanied by a narrowed disc space in the control and TGF‐β1‐inhibitor injection groups at 8 weeks after injection, but there was no significant difference between the two groups. However, the T2‐weighted images of the discs in the PRP group exhibited higher signal intensity and wider disc space than those in the other two groups, which was similar to the histological and immunohistochemical analysis findings. Haematoxylin and eosin staining showed disordered discs and no nucleus pulposus tissue regeneration in the control or TGF‐β1 inhibitor injection groups. However, the structure of the outer layers of the peripheral annulus tissue was normal in the PRP group and regeneration was observed in the nucleus pulposus, which was further validated by an increase in collagen II found using immunohistochemical analysis. We further confirmed the role of PRP *in vivo*. Compared with the control group, the PRP group showed a significant increase in Smad2/3 according to immunohistochemical staining. This suggested that PRP could activate the biological activity of Smad2/3, which effectively promotes the synthesis and secretion of collagen II and other matrix components that contribute to the structural and functional recovery of intervertebral disc degeneration.

## Conclusion

In summary, PRP is rich in growth factors, especially TGF‐β1, which can enhance the nucleus pulposus cells proliferation and anabolic processes *via* the TGF‐β1/Smad2/3 pathway. Platelet‐rich plasma injections could also retard intervertebral disc degeneration in a rabbit model. Elucidating the mechanism of PRP therapy would provide valuable information for the clinical treatment of intervertebral disc degeneration.

## Conflicts of interest

The authors declare no conflict of interest.
